# Serial Mediation Pathways Linking Physical Performance to EQ-VAS-Assessed Self-Rated Health in Older Inpatients

**DOI:** 10.3390/healthcare14203390

**Published:** 2026-10-12

**Authors:** Ovidiu Lucian Băjenaru, Lidia Băjenaru, Alexandru Balog, Gabriela Cristina Chelu, Alexandru Constantinescu, Octavian Andronic, Cătălina Raluca Nuță

**Affiliations:** 1 Faculty of Medicine, “Carol Davila” University of Medicine and Pharmacy, 050474 Bucharest, Romania; ovidiu.bajenaru@umfcd.ro (O.L.B.); alexandru.constantinescu@umfcd.ro (A.C.); octavian.andronic@umfcd.ro (O.A.); catalina.nuta@umfcd.ro (C.R.N.); 2 National Institute of Gerontology and Geriatrics “Ana Aslan”, 011241 Bucharest, Romania; 3 Department of Communications, Applications, and Digital System, National Institute for Research and Development in Informatics—ICI Bucharest, 011455 Bucharest, Romania; 4 Department of Computer Science, Faculty of Automatic Control and Computers, National University of Science and Technology POLITEHNICA Bucharest, 060042 Bucharest, Romania; 5 Doctoral School of Economic Informatics, Bucharest University of Economic Studies, 010374 Bucharest, Romania; sandu.balog@gmail.com; 6 Gastroenterology Department, University Emergency Hospital, 050098 Bucharest, Romania; 7 Center of Innovation and e-Health, “Carol Davila” University of Medicine and Pharmacy, 030167 Bucharest, Romania

**Keywords:** physical performance, balance and gait, depressive symptoms, cognitive status, functional status, self-rated health, EQ-VAS, older adults

## Abstract

**Background/Objectives:** This study examined patterns of cross-sectional indirect associations involving cognitive status, depressive symptoms, and functional status in the relationship between Tinetti Mobility Test (TMT)-assessed balance and gait performance and self-rated health assessed with the EuroQol Visual Analogue Scale (EQ-VAS) in older inpatients. **Methods**: The study included 287 Romanian inpatients aged ≥65 years. Depressive symptoms, cognitive status, and functional status were assessed using the 15-item Geriatric Depression Scale (GDS-15), Mini-Mental State Examination (MMSE), and Lawton Instrumental Activities of Daily Living (IADL) scale, respectively. Mediation analysis was performed using the PROCESS macro v4.3.1 for SPSS. Model 80 was used with 5000 bootstrap samples and adjusted for sex, age, residence, and obesity. **Results**: Compared with participants in the impaired balance and gait group (scores 0–23), those in the unimpaired balance and gait group (scores 24–28) showed positive indirect associations with EQ-VAS scores through depressive symptoms (B = 0.540, Bootstrap 95% CI: [0.170, 1.064]), cognitive status (B = 0.557, Bootstrap 95% CI: [0.048, 1.373]), and functional status (B = 0.796, Bootstrap 95% CI: [0.218, 1.510]), and through serial pathways involving depressive symptoms followed by IADL (B = 0.067, Bootstrap 95% CI: [0.001, 0.191]) and cognitive status followed by IADL (B = 0.224, Bootstrap 95% CI: [0.016, 0.554]). **Conclusions**: TMT-assessed balance and gait performance was associated with EQ-VAS-assessed self-rated health through several model-specified psychological, cognitive, and functional pathways. These cross-sectional, model-dependent findings do not establish temporal ordering or causal mediation and apply to global self-rated health rather than the full multidimensional construct of health-related quality of life.

## 1. Introduction

Population ageing has become a major public health concern worldwide. In the European Union (EU), adults aged 65 years and older accounted for approximately 21% of the population in 2020, and this proportion is projected to approach 29% by 2050. Romania is experiencing a similar demographic transition, with the share of people aged 65 years and older expected to reach 27.7% by 2050 [1]. At older ages, chronic disease, impaired mobility, functional limitations, and cognitive decline frequently coexist and may substantially compromise health-related quality of life (HRQOL). Because HRQOL reflects physical, psychological, social, and functional dimensions of health, identifying modifiable factors associated with it—and clarifying how these factors are related—is important for designing interventions that respond to the needs of older patients.

Physical performance is one such factor, defined in this study as an individual’s maximum capacity to perform a specific physical task. However, the definitions and operationalization of mobility and physical performance vary across studies [2,3]. Their clinical relevance extends beyond detecting mobility impairment, as reduced physical performance is also associated with loss of independence, greater care needs, and poorer perceived health.

A substantial body of research has documented associations between physical performance, mobility-related factors, and HRQOL in older adults [4,5,6,7,8,9]. Sunde et al. found that better physical function, assessed using the Short Physical Performance Battery (SPPB), was associated with higher scores on the physical dimensions of the SF-36 among older adults with, or at risk of, mobility disability after hospital discharge [4]. Studies have also reported positive associations between standing balance, walking speed, handgrip strength, and WHOQOL-BREF scores [5]. Likewise, Lechuga Azdar et al. observed that low SPPB performance was associated with lower EQ-VAS-rated health in community-dwelling older adults [6]. Among older adults with cognitive impairment, Song et al. identified grip strength, balance, and mobility as significant correlates of HRQOL [7]. Evidence from a Romanian inpatient sample of 359 patients showed associations between functional performance measures, including the Tinetti Mobility Test (TMT) and the Up and Go Test, and the EQ-5D-5L dimensions. In particular, higher TMT scores, indicating better balance and gait performance, were strongly correlated with fewer reported problems in the mobility and self-care dimensions across age groups [10].

Taken together, these studies consistently link better physical performance with better perceived health and quality of life [4,5,6,7]. They do not, however, fully explain the psychological, cognitive, and functional pathways through which this relationship may operate.

This distinction is important because HRQOL is shaped by more than physical ability alone. A systematic review of 26 studies identified individual, psychosocial, psychological, and functional characteristics as relevant determinants of active aging and quality of life [11]. Among these characteristics, cognitive status, depressive symptoms, and functional independence are particularly relevant to geriatric care. Each is associated with physical performance and HRQOL, but their combined role within a single explanatory model remains insufficiently understood.

Cognitive function comprises interrelated abilities such as memory, reasoning, judgment, attention, and executive control. Decline in these abilities can affect planning, task initiation, medication and financial management, adherence to clinical recommendations, safe mobility, and the performance of complex daily activities. Song et al. showed that cognitive function mediated the association between physical function—measured through grip strength, balance, and mobility—and the physical and mental components of the SF-36 in older adults with mild cognitive impairment [7]. Demirhan et al. [12] similarly reported that poorer cognitive functioning was associated with lower mental HRQOL in older patients and that depressive symptoms assessed using the 15-item Geriatric Depression Scale [13] contributed to this association. These findings suggest a direct relationship between cognitive status and perceived health, as well as an indirect connection through the interplay of affective and functional factors. Nevertheless, cognition has rarely been examined as an intermediate variable between physical performance and HRQOL within a broader multiple-mediation framework.

Depressive symptoms represent a second plausible pathway [14]. They are common in later life and may adversely affect HRQOL through reduced motivation, hopelessness, social withdrawal, and diminished engagement in everyday activities. Previous studies have documented direct negative associations between depressive symptoms and HRQOL [15,16], as well as indirect effects involving performance status [17], balance self-efficacy [18], and physical activity [9]. For instance, a lower burden of depressive symptoms partly explains the association between various types of physical activity and HRQOL [9]. Higher SPPB scores have also been associated with a lower likelihood of depressive symptoms [19]. Thus, the relationship between reduced physical performance and poorer HRQOL may reflect not only functional restriction but also mood-related vulnerability, including reduced confidence and participation.

Functional status provides a further link between physical, cognitive, and psychological health. It refers to an individual’s ability to live independently and is commonly evaluated through activities of daily living (ADL) and instrumental activities of daily living (IADL). IADL encompass complex tasks required for community living, including shopping, meal preparation, housekeeping, transportation, medication management, and financial management. Successful performance of these activities depends on mobility, executive function, motivation, emotional health, and social participation. Greater independence is generally associated with better quality of life, whereas functional decline is associated with increased dependency, poorer health, and greater care requirements.

Austrian data illustrate the public health importance of functional decline: the adjusted odds of IADL limitations were 94% higher in 2019 than in 2014 [20]. In a systematic review of 83 studies, 62 articles reported physical-performance measures; low physical performance was associated with ADL dependency in 37 of 49 studies and with IADL dependency in 9 of 11 studies [21]. Functional status has also been associated with physical function and frailty-related physical indicators [22,23,24], physical activity [25,26], cognitive function [27,28,29], and quality of life [27,28,29,30]. More recent studies have treated ADL, IADL, or other disability-related functional measures as intermediate variables connecting cognitive, psychological, and health-related factors [31,32,33].

From a clinical perspective, this suggests that interventions focused exclusively on mobility may be incomplete if cognitive status, depressive symptoms, and the capacity for independent living are not considered concurrently.

Despite this growing evidence, several questions remain unresolved. Much of the available research has primarily focused on Western European or Asian populations, leaving limited evidence from Central and Eastern Europe, including Romania. Moreover, studies have commonly examined pairwise associations or models with a single mediator, rather than evaluating how psychological, cognitive, and functional factors may operate together. The role of functional status as an intermediate variable linking physical and cognitive vulnerability to HRQOL is still insufficiently characterized. Evidence is particularly scarce for Romanian older adults assessed with standardized measures of physical performance, depressive symptoms, cognition, functional independence, and HRQOL within the same analytical framework.

The present study therefore examined statistical indirect associations between TMT-assessed balance and gait performance and global self-rated health assessed with the EQ-VAS through depressive symptoms, cognitive status, and functional status among Romanian geriatric inpatients aged ≥65 years. A serial multiple-mediation model was specified in which depressive symptoms and cognitive status were included as parallel first-stage mediators, followed by functional status as a second-stage mediator. This structure allowed the mediating role of each variable to be assessed separately, together with the serial pathways involving depressive symptoms or cognitive status followed by functional status.

The conceptual specification of the present analysis was informed by the revised Wilson and Cleary model of health-related quality of life proposed by Ferrans et al. [34]. This framework distinguishes symptoms, functional status, general health perceptions, and overall quality of life, while further conceptualizing functional status in terms of functional capacity and functional performance. Within the present study, TMT-assessed balance and gait performance was considered an indicator of physical functional capacity, depressive symptoms assessed by the GDS-15 represented the symptom domain, and MMSE performance reflected cognitive functional capacity. IADL was conceptualized as functional performance because it captures the ability to carry out complex activities required for independent daily living. Finally, the EQ-VAS was mapped to general health perceptions, as it provides a global subjective evaluation of current health rather than a comprehensive measure of overall quality of life.

This framework provides a conceptual rationale for positioning depressive symptoms and cognitive status before IADL in the specified analytical model: emotional symptoms and cognitive capacity may be associated with the extent to which individuals are able to perform complex daily activities, while functional performance may, in turn, be reflected in their global perception of health. Accordingly, GDS-15 and MMSE were specified as parallel first-stage mediators and IADL as a subsequent mediator. The framework was used to inform the analytical ordering rather than to assert temporal precedence. Because all variables were assessed cross-sectionally, reciprocal and alternative relationships remain possible.

Based on this theoretical framework and the empirical evidence reviewed above, the following theory-informed directional hypotheses were evaluated:

**H1a.**  *Compared with participants in the impaired balance and gait group (scores 0–23), those in the unimpaired balance and gait group (scores 24–28) would show positive indirect associations with EQ-VAS scores through fewer depressive symptoms.*

**H1b.**  *Compared with participants in the impaired balance and gait group, those in the unimpaired balance and gait group would show positive indirect associations with EQ-VAS scores through better cognitive status.*

**H1c.**  *Compared with participants in the impaired balance and gait group, those in the unimpaired balance and gait group would show positive indirect associations with EQ-VAS scores through greater independence in instrumental activities of daily living.*

**H2a.**  *Compared with participants in the impaired balance and gait group, those in the unimpaired balance and gait group would show a positive serial indirect association with EQ-VAS scores through fewer depressive symptoms followed by greater IADL independence.*

**H2b.**  *Compared with participants in the impaired balance and gait group, those in the unimpaired balance and gait group would show a positive serial indirect association with EQ-VAS scores through better cognitive status followed by greater IADL independence.*

To our knowledge, few studies have jointly examined these psychological, cognitive, and functional domains within a single mediation model in a geriatric inpatient sample. By examining these interrelationships, the study aimed to provide a clinically integrated account of factors associated with HRQOL and to inform the early identification, targeted assessment, and follow-up of vulnerable older adults. Given the cross-sectional design, the proposed mediation pathways were interpreted as statistical associations rather than temporal or causal mechanisms.

Unlike previous analyses from the parent cohort, the present exploratory secondary analysis jointly examined depressive symptoms, cognitive status, and functional status as potential statistical mediators of the association between physical performance and EQ-VAS-assessed self-rated health.

## 2. Materials and Methods

### 2.1. Study Design and Participants

This cross-sectional study utilized data from a larger study on health-related quality of life conducted in Romania. The study recruited patients who were consecutively admitted to the National Institute of Gerontology and Geriatrics “Ana Aslan”, Bucharest, between January 2024 and April 2025 [10]. Each eligible patient received an explanation of the study aims and procedures and provided written informed consent before enrolment; patients who did not provide consent were excluded. The parent cohort comprised 359 inpatients aged ≥40 years. For the present secondary analysis, 72 participants aged 40–64 years were excluded. All remaining 287 participants aged ≥65 years had complete data for TMT, GDS-15, MMSE, IADL, EQ-VAS, and the included covariates and were retained in the analysis. No missing data were recorded, and no imputation procedures were required. The parent study received initial approval from the Ethics Committee of the National Institute of Gerontology and Geriatrics “Ana Aslan”, Bucharest, under Protocol Code 113 on 23 November 2023, before recruitment and data collection began in January 2024. Protocol Code 231, issued on 4 April 2025 during the final month of the reported data-collection period, represented a subsequent approval related to the continuation and administrative documentation of the same study. The initial authorization under which data collection commenced was therefore Protocol Code 113. The study was conducted in accordance with the ethical principles of the Declaration of Helsinki and applicable national regulations. Data from the same parent cohort have previously been used in secondary analyses addressing quality of life and functional and nutritional impairment [10], motor and functional correlates of cognitive performance [35], and frailty-related cardiovascular, metabolic, and renal characteristics [36]. All 287 participants included in the present study constitute the subgroup aged ≥65 years drawn from the same parent cohort of 359 inpatients; therefore, there is direct participant overlap with these previous publications. There is also overlap in several study measures: TMT and EQ-VAS were examined in [10]; TMT, MMSE, and IADL were included in [35]; and TMT and MMSE were included in [36]. However, GDS-15 was not analysed in any of these previous studies, and none of them jointly examined TMT, depressive symptoms, cognitive status, functional status, and EQ-VAS within a serial multiple-mediation model. The earlier publications addressed different research questions using descriptive, correlational, regression, or ROC analyses. The present mediation analysis was not prespecified in the original parent-study protocol and is therefore reported as a secondary exploratory analysis. Its distinct objective was to examine the statistical indirect associations among these variables in hospitalized adults aged ≥65 years. All study measures were obtained during the same assessment session in accordance with the standardized study protocol.

Because the present investigation was a secondary exploratory analysis of an existing cohort, no prospective sample-size calculation was performed specifically for the serial multiple-mediation model. The analytic sample was determined by the number of participants aged 65 years or older who met the eligibility criteria and had complete data for all model variables and included covariates. Accordingly, all 287 eligible participants were retained in the analysis.

### 2.2. Measures

#### 2.2.1. Physical Performance

Physical performance was assessed using the Tinetti Mobility Test (TMT), also known as the Performance-Oriented Mobility Assessment, which evaluates balance and gait performance on a total scale ranging from 0 to 28, with higher scores indicating better performance [37,38,39]. Scores of 24–28 correspond to the conventionally classified low-fall-risk range, scores of 19–23 to moderate fall risk, and scores of 0–18 indicate high fall risk. For the present study, the TMT moderate- and high-fall-risk categories were combined to provide a clinically interpretable contrast between participants with the low-fall-risk scores (24–28; “unimpaired balance and gait”) and those scoring below these scores (0–23; “impaired balance and gait”).

#### 2.2.2. Depressive Symptoms

Depressive symptoms were assessed using the 15-item Geriatric Depression Scale (GDS-15) [13], with total scores ranging from 0 to 15, with a higher score indicating a higher level of depression in older people. Scores of 0–4 are considered normal, 5–8 indicate mild depression, 9–11 indicate moderate depression, and 12–15 indicate severe depression.

#### 2.2.3. Cognitive Status

Cognitive status was assessed using the Mini-Mental State Examination (MMSE) on a scale of 0 to 30 points [40]. Higher scores indicate better cognitive performance. MMSE scores of ≥27 indicate normal, scores of 21–26 indicate mild cognitive impairment, scores of 11–20 indicate moderate cognitive impairment, and scores of ≤10 indicate severe cognitive impairment.

#### 2.2.4. Functional Status

Functional status was assessed using the Instrumental Activities of Daily Living (IADL) scale. The IADL assesses the ability to perform functional activities essential for independent living, including telephone use, shopping, food preparation, housekeeping, laundry, transportation, medication management, and financial handling. The total score ranges from 0 (low function, dependent) to 8 (high function, independent) [41,42].

#### 2.2.5. Health-Related Quality of Life

The EQ-5D-5L comprises two components: a descriptive system covering mobility, self-care, usual activities, pain/discomfort, and anxiety/depression, and the EuroQol Visual Analogue Scale (EQ-VAS) [43]. The present analysis used only the EQ-VAS component of the Romanian EQ-5D-5L; neither the five descriptive dimensions nor an EQ-5D-5L utility index was analysed as an outcome. The EQ-VAS records participants’ overall self-rated health on the day of assessment on a scale ranging from 0 (the worst health imaginable) to 100 (the best health imaginable). Accordingly, the outcome examined in this study is interpreted as global self-rated health assessed with the EQ-VAS, rather than as a comprehensive measure of all dimensions of HRQOL. Romanian population norms for the EQ-5D-5L, including the EQ-VAS, have been reported previously [44].

#### 2.2.6. Covariates

Covariates were selected on the basis of substantive clinical considerations and their availability and completeness in the parent dataset, rather than solely on statistical significance in univariable analyses. The model was adjusted for sex (0 = female, 1 = male), age categorized into three distinct groups (65–74 years, 75–79 years, and ≥80 years), residence area (0 = urban, 1 = rural), and obesity (0 = no, 1 = yes). Sex and age were included as core demographic covariates plausibly related to physical, cognitive, and functional status; residence area was included as a contextual indicator that may reflect differences in living environment and access to care; and obesity was included as an anthropometric condition plausibly associated with mobility and self-rated health.

### 2.3. Data Analysis

Data were analysed using SPSS version 24.0. Descriptive statistics were used to describe demographic and clinical characteristics of patients, as well as variables of interest in the mediation model. Categorical variables were reported as frequencies and percentages. Continuous variables were reported as means (M) and standard deviations (SD). Correlations between variables were assessed using Spearman’s rank correlation coefficient.

**Figure 1:**
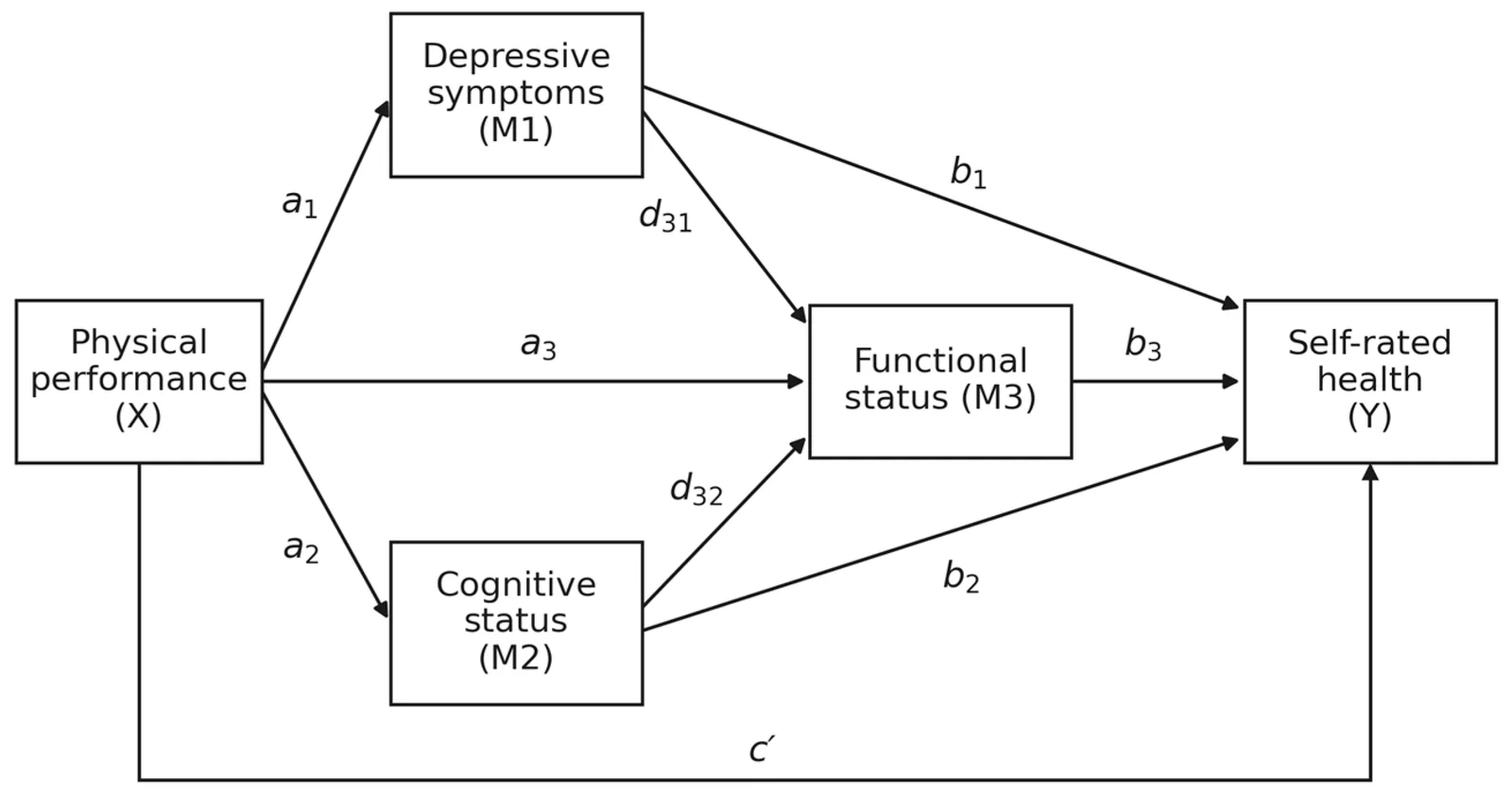
Diagram of the hypothesized serial multiple mediation model.

The mediation analysis was conducted using the PROCESS macro v4.3.1 for SPSS, developed by Andrew F. Hayes [45]. Specifically, model 80 was applied to examine relationships involving multiple mediators that were linked sequentially. Figure 1 shows the hypothesized mediation pathway model that was proposed. Model 80 was selected as the primary analytical specification because it corresponded to the theory-informed conceptual ordering described in the Introduction, in which mobility-related performance was positioned upstream of affective and cognitive functioning and of independence in complex daily activities.

In this study, we examined whether TMT-assessed balance and gait performance was indirectly associated with EQ-VAS-assessed self-rated health through two parallel mediators—depressive symptoms and cognitive status—and subsequently through functional status within the specified serial mediation model. The model includes nine direct paths (c’, a1, a2, a3, d31, d32, b1, b2, and b3) and five indirect effects (a1–b1, a2–b2, a3–b3, a1–d31–b3, and a2–d32–b3). The five specific indirect associations estimated with Model 80 corresponded to hypotheses H1a–H1c and H2a–H2b formulated in the Introduction. Indirect associations were estimated using 5000 bootstrap resamples and are reported with 95% bootstrap confidence intervals to convey the precision and uncertainty of the estimates. An indirect association was considered statistically different from zero when its 95% bootstrap confidence interval did not include zero. Throughout this manuscript, the terms “direct effect” and “indirect effect” are used as conventional statistical labels for the corresponding PROCESS model estimates and are interpreted as cross-sectional associations rather than causal effects.

The direct effect (*c’*) refers to the relationship between TMT and EQ-VAS after adjustment for the mediators. In chain mediation analysis, the specific indirect effects are model-estimated associations between TMT and EQ-VAS through GDS-15 (a1*b1), through MMSE (a2*b2), through IADL (a3*b3), or through GDS-15 and IADL (a1*d31*b3), as well as through MMSE and IADL (a2*d32*b3). The total effect (*c*) refers to the relationship between TMT and EQ-VAS without controlling for mediators (GDS-15, MMSE, and IADL).

For the primary analysis, the TMT moderate- and high-fall-risk categories were combined to provide a clinically interpretable contrast between participants in the low-fall-risk range (24–28; labeled “unimpaired balance and gait” and coded as 1) and those scoring below this range (0–23; labeled “impaired balance and gait” and coded as 0). Because categorization reduces score information [46], the TMT total score was also analysed as a continuous predictor in a sensitivity analysis. To address the negative skewness of the original score, it was reflected and log-transformed before inclusion in Model 80. Results are provided in the Supplementary Material, Table S1.

As a sensitivity analysis, we estimated an exploratory alternative specification (PROCESS Model 81 [45]), in which IADL preceded GDS-15 and MMSE. This analysis was used to examine whether other cross-sectional pathway structures were also compatible with the observed data; it was not used to determine a correct temporal or causal ordering among the mediators. Results are provided in the Supplementary Material, Table S2.

For reporting, standardized regression coefficients (β) are presented in the path diagram, whereas unstandardized effects (B) with bootstrap confidence intervals are reported for the total, direct, and specific indirect effects. All models were adjusted for the possible confounders of sex, age, residence area, and obesity. Given the cross-sectional design, all mediation pathways are interpreted as statistical indirect associations and do not establish causal pathways or temporal ordering. Preliminary analyses were conducted before main analyses. Multicollinearity tests showed correlations lower than 0.60, and variance inflation factor (VIF) values ranged between 1.099 and 1.797. Moreover, to avoid possible effects of heteroskedasticity on the validity of inference, HC4—a heteroskedasticity-consistent estimator—was used [45].

## 3. Results

### 3.1. Demographic and Clinical Characteristics

A total of 287 patients aged ≥65 years were included in this study. The mean age was 74.8 years (standard deviation = 6.385). The largest age group was 65–74 years (50.5%), followed by participants aged 75–79 years (26.1%). The patients were predominantly female (79.8%). Most participants resided in urban areas (70.4%) compared to rural settings (29.6%), and 33.8% had obesity. Additional characteristics are provided in Table 1.

### 3.2. Descriptive Statistics and Correlation Analysis

The descriptive statistics and Spearman correlations of variables of interest in the mediation model (TMT, GDS-15, MMSE, IADL, and EQ-VAS) are presented in Table 2. The mean physical performance score, assessed using the Tinetti Mobility Test, was 23.3 (SD = 5.04), with 52.3% of participants showing normal balance and gait. Most participants had normal cognitive status (78.4%), whereas 21.6% had some degree of cognitive impairment. The IADL score had a mean of 7.27 (SD = 1.41), suggesting that the studied sample had a relatively high level of independence in instrumental activities of daily living. Finally, the mean EQ-VAS score was 86.9 (SD = 7.25), suggesting that participants reported high self-rated health status.

Correlation analysis revealed significant associations among variables. Specifically, physical performance was negatively associated with depressive symptoms and positively associated with other variables. Depressive symptoms were significantly and negatively associated with all other variables. All other correlations were positive and statistically significant. The highest correlation (0.58) was between physical performance and functional status.

### 3.3. Regression Analysis

**Figure 2:**
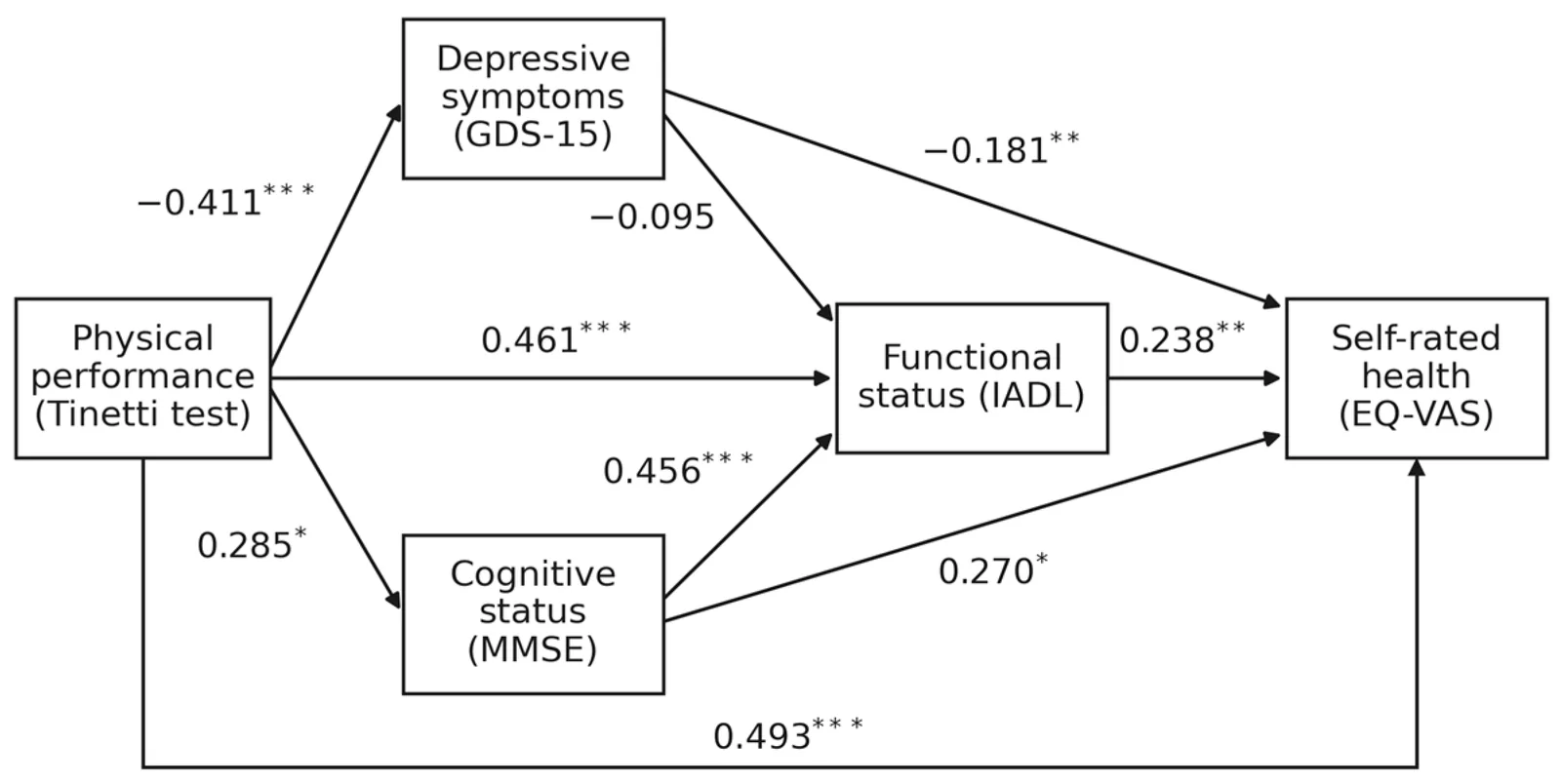
Chain mediation diagram (* *p* < 0.05, ** *p* < 0.01, *** *p* < 0.001).

In regression analysis, physical performance assessed by the Tinetti mobility test (TMT) was analysed as a categorical variable: “impaired balance and gait” (coded as 0) and “unimpaired balance and gait” (coded as 1). Depressive symptoms (GDS-15), cognitive status (MMSE), functional status (IADL), and global self-rated health (EQ-VAS) were analysed as continuous dependent variables in their respective regression models. The regression models were adjusted for sex, age, residence area, and obesity (see Figure 2 and Table 3). Coefficients for continuous variables are fully standardized. Coefficients for the dichotomous predictor (TMT) are partially standardized, reflecting the change in standard deviations of the outcome variable between the two TMT groups.

Compared with participants in the impaired balance and gait performance group, those in the unimpaired balance and gait performance group had lower GDS-15 scores (β = −0.411, t = −3.513, *p* < 0.001), higher MMSE scores (β = 0.285, t = 2.228, *p* = 0.027), and higher IADL scores (β = 0.461, t = 5.125, *p* < 0.001). MMSE was positively associated with IADL (β = 0.456, t = 4.135, *p* < 0.001), whereas the GDS-15 → IADL component path did not reach the conventional *p* < 0.05 threshold (β = −0.095, t = −1.745, *p* = 0.082). The complete IADL regression model explained 48.6% of the variance.

In the model including TMT and the covariates, unimpaired balance and gait performance was positively associated with EQ-VAS scores (β = 0.794, t = 7.174, *p* < 0.001), and the model accounted for 20.8% of the variance. After adding GDS-15, MMSE, and IADL, the association between unimpaired balance and gait performance on the TMT and EQ-VAS remained positive (β = 0.493, t = 5.118, *p* < 0.001). MMSE (β = 0.270, t = 2.414, *p* = 0.016) and IADL (β = 0.238, t = 2.743, *p* = 0.006) were positively associated with EQ-VAS, whereas GDS-15 was negatively associated with EQ-VAS (β = −0.181, t = −2.886, *p* = 0.004). The full model accounted for 41.5% of the variance.

### 3.4. Analysis of Indirect Association Patterns

We conducted a mediation analysis using PROCESS Model 80 to evaluate the indirect associations within our model. Specifically, the results indicated that compared to the impaired balance and gait performance group, the unimpaired balance and gait performance group exhibited a significantly positive direct association with EQ-VAS (B = 3.576, 95% CI: [2.200, 4.952]). In the examination of total association, the unimpaired balance and gait performance group demonstrated a significantly positive total association with EQ-VAS compared to the impaired balance and gait performance group (B = 5.761, 95% CI: [4.180, 7.341]). More details are presented in Figure 2 and Table 4.

The analysis further examined each of the multiple modelled indirect pathways. In Path 1 (TMT → GDS-15 → EQ-VAS) and Path 2 (TMT → MMSE → EQ-VAS), in comparison to the impaired balance and gait performance group, the unimpaired balance and gait performance group exhibited a significantly positive specific indirect association with EQ-VAS through the mediating pathway of depressive symptoms (B = 0.540, Bootstrap 95% CI: [0.170, 1.064]; higher GDS-15 score indicates greater severity of depression) and through the mediating pathway of cognitive status (B = 0.557, Bootstrap 95% CI: [0.048, 1.373]). Moving to Path 3 (TMT → IADL → EQ-VAS), in comparison to the impaired balance and gait performance group, the unimpaired balance and gait performance group had a significantly positive specific indirect association with EQ-VAS through the mediating role of functional status (B = 0.796, Bootstrap 95% CI: [0.218, 1.510]). These findings were consistent with the theory-informed hypotheses H1a–H1c under the specified cross-sectional model.

In Path 4 (TMT → GDS-15 → IADL → EQ-VAS), compared to the reference group, the unimpaired balance and gait performance group displayed a significantly positive specific indirect association with EQ-VAS through the serial mediation of depressive symptoms and functional status (B = 0.067, Bootstrap 95% CI: [0.001, 0.191]). In Path 5 (TMT → MMSE → IADL → EQ-VAS), compared to the reference group, the unimpaired balance and gait performance group displayed a significantly positive specific indirect association with EQ-VAS through the serial mediation of cognitive status and functional status (B = 0.224, Bootstrap 95% CI: [0.016, 0.554]). The serial indirect association through depressive symptoms and functional independence was small (Ind4: B = 0.067, BootSE = 0.050), and its 95% bootstrap confidence interval narrowly excluded zero [0.001, 0.191]. The GDS-15 → IADL component path did not reach the conventional *p* < 0.05 threshold (β = −0.095, *p* = 0.082); however, inference for Ind4 concerns the bootstrap distribution of the product of the component coefficients and is not conditional on each individual path being statistically significant. Because the lower confidence limit was very close to zero, this indirect association should be interpreted cautiously. These primary-model results were consistent with H2a and H2b, with the association corresponding to H2a interpreted cautiously.

Pairwise contrasts between specific indirect associations (Table 4) showed that Ind1 (TMT → GDS-15 → EQ-VAS) had a larger estimate than Ind4 (TMT → GDS-15 → IADL → EQ-VAS; difference = 0.473, 95% bootstrap CI [0.117, 0.983]). Ind3 (TMT → IADL → EQ-VAS) had a larger estimate than both Ind4 (difference = 0.729, 95% bootstrap CI [0.181, 1.403]) and Ind5 (TMT → MMSE → IADL → EQ-VAS; difference = 0.572, 95% bootstrap CI [0.084, 1.232]). The 95% bootstrap confidence intervals for the remaining seven pairwise contrasts included zero.

When a reflected log-transformed TMT-derived predictor was entered as a continuous variable, four of the five specific indirect associations had 95% bootstrap confidence intervals excluding zero. The exception was the serial indirect association through depressive symptoms and IADL (Ind4: B = −0.0583, 95% bootstrap CI [−0.2089, 0.0304]; Supplementary Table S1). Because the interval for Ind4 included zero in this sensitivity analysis, the corresponding finding from the primary binary-TMT analysis warrants cautious interpretation. As an exploratory alternative-specification analysis, we estimated PROCESS Model 81 [45], in which IADL preceded GDS-15 and MMSE. Four of the five specific indirect effects had bootstrap 95% confidence intervals that excluded zero: TMT → IADL → EQ-VAS (B = 1.088, bootstrap 95% CI [0.301, 2.005]), TMT → GDS-15 → EQ-VAS (B = 0.368, bootstrap 95% CI [0.053, 0.810]), TMT → IADL → GDS-15 → EQ-VAS (B = 0.172, bootstrap 95% CI [0.037, 0.378]), and TMT → IADL → MMSE → EQ-VAS (B = 0.685, bootstrap 95% CI [0.164, 1.497]). The specific indirect association through MMSE alone was not statistically significant (B = −0.128, 95% CI [−0.601, 0.297]). Thus, the alternative model also yielded several indirect associations whose confidence intervals excluded zero, indicating that multiple cross-sectional pathway specifications are compatible with the observed data. Results are provided in Supplementary Table S2. This analysis does not establish the temporal ordering of the mediators or validate Model 80 as the uniquely correct specification.

## 4. Discussion

This study examined the cross-sectional indirect association patterns involving cognitive status, depressive symptoms, and functional status in the relationship between physical performance measured using the Tinetti mobility test (TMT) and global self-rated health assessed with the EQ-VAS among Romanian older inpatients. The mediation analysis was conducted using the PROCESS macro v4.3.1 for SPSS (Model 80) [45]. Three single-mediator and two serial indirect associations had bootstrap confidence intervals that excluded zero. These findings describe model-dependent statistical patterns and do not establish temporal ordering or causal mediation.

This research represents an innovative exploratory study on the associations among physical performance, cognitive status, depressive symptoms, functional status, and self-rated health in older adults in a serial multiple-mediation model. The research yielded several notable findings. First, physical performance (TMT), cognitive status (MMSE), depressive symptoms (GDS-15), and functional performance (IADL) were significantly associated with self-rated health (EQ-VAS). Second, the specified model yielded indirect associations between TMT-assessed balance and gait performance and EQ-VAS-assessed self-rated health through depressive symptoms, cognitive status, and functional status. Third, the mediation analysis showed that relationships between physical performance and self-rated health yielded serial indirect associations under the specified model through cognitive status and functional status, as well as through depressive symptoms and functional status. These findings extend beyond previous studies by providing a comprehensive framework that integrates multiple pathways linking physical performance to self-rated health.

### 4.1. Direct Association Between Physical Performance and Self-Rated Health

We observed a significant association between physical performance (balance, gait, and mobility) and EQ-VAS scores among older inpatients. Better physical performance was associated with higher EQ-VAS scores. Conversely, lower physical performance is commonly associated with greater dependency, lower quality of life, and greater care needs. After adjustment for sex, age, residence area, and obesity, participants in the unimpaired balance and gait group had higher EQ-VAS scores than those in the impaired group. The model including TMT and these covariates accounted for 20.8% of the variance in EQ-VAS scores (Table 3). This association is consistent with previous studies in several countries using different measurement instruments linking physical performance with perceived health or multidimensional HRQOL [4,5,6,47], although the present outcome specifically reflects global self-rated health measured with the EQ-VAS. In Norway, better physical function measured with the Short Physical Performance Battery (SPPB) was significantly associated with a higher score on the physical dimensions of the SF-36 (HRQOL) in older adults with or at risk of mobility disability [4]. In Korea, mobility was associated with both physical and mental components of the SF-36 (HRQOL) among older adults with cognitive impairment [7]. A study among Polish post-stroke inpatients showed that worse balance and gait performance on the Tinetti test were strongly associated with lower SF-36 scores [47]. A study of community-dwelling older Mexicans reported that low physical performance assessed with SPPB was associated with lower EQ-VAS scores [6].

### 4.2. Simple Indirect Association Patterns

The specified model yielded a statistically significant specific indirect association between physical performance and EQ-VAS through depressive symptoms. Better physical performance was associated with fewer depressive symptoms, which, in turn, was associated with higher EQ-VAS scores. This result is in line with previous studies demonstrating the mediating role of depressive symptoms in different contexts, such as the relationship between physical capability and quality of life among stroke patients [18] as well as the relationship between the types of physical activity and HRQOL among older adults [9,48].

The model also yielded a positive specific indirect association through cognitive status. Better physical performance was associated with higher cognitive status, which, in turn, was associated with higher EQ-VAS scores. This result is consistent with previous studies demonstrating the mediating role of cognitive status in different contexts, such as the relationship between physical function and quality of life among older adults [7] as well as the relationship between the types of physical activity and frailty risk [49].

Similarly, the specified model yielded a positive specific indirect association between physical performance and EQ-VAS through functional status. Better physical performance was associated with greater functional independence, which, in turn, was associated with higher EQ-VAS scores. This result is consistent with previous studies demonstrating the mediating role of ADL/IADL in different contexts, such as the relationship between intrinsic capacity [2] and HRQOL among community-dwelling and institutionalized adults aged 60 years and older [33], the relationship between sleep quality and HRQOL among older people [31], as well as the relationship between multimorbidity and HRQOL among the Chinese elderly [50].

Given the cross-sectional design, these patterns are compatible with, but do not demonstrate, the proposed conceptual ordering and should be interpreted as statistical indirect associations rather than as evidence of a causal sequence.

### 4.3. Multiple Indirect Association Patterns

Serial mediation pathway analysis showed that, under the specified cross-sectional model, physical performance was indirectly associated with EQ-VAS-assessed self-rated health through cognitive status and IADL, and through depressive symptoms and IADL.

In the primary binary-TMT model, the TMT → GDS-15 → IADL → EQ-VAS pathway yielded a small positive serial indirect association. The GDS-15 → IADL component path did not reach the conventional *p* < 0.05 threshold (β = −0.095, *p* = 0.082). This does not invalidate inference on the bootstrapped product, but the confidence interval for Ind4 narrowly excluded zero and included zero when a reflected log-transformed continuous TMT-derived predictor was used (Supplementary Table S1). This pathway should therefore be interpreted cautiously, as its statistical support depends on the predictor specification. The TMT → MMSE → IADL → EQ-VAS pathway also yielded a positive serial indirect association under Model 80. These concurrent, model-dependent findings do not establish temporal ordering or causal mechanisms. Longitudinal studies are needed to evaluate the proposed sequence.

In summary, our study advances the current knowledge about the complex relationships among physical performance, cognitive status, depressive symptoms, functional status, and self-rated health. While earlier research has explored pairwise associations between factors [4,19,27], simple mediation models [7,8,12,33,48] or serial multiple mediator models with only two mediators [18,19], our study uniquely explores multiple modeled indirect pathways. Importantly, these findings suggest that psychological, cognitive, and functional factors form an interrelated pattern of cross-sectional associations with self-rated health among older inpatients. The observed pattern is conceptually compatible with the revised Wilson and Cleary framework proposed by Ferrans et al. [34], particularly its distinction between symptoms, functional capacity, functional performance, and general health perceptions. This theoretical correspondence informed the model specification but does not validate the temporal direction of the paths.

Given the cross-sectional nature of the data in our study, an alternative model of the observed relationships was also considered. Although the specified model assigns directions to the paths, the cross-sectional data are also compatible with other path directions. Model 81, in which IADL preceded GDS-15 and MMSE, was examined as an exploratory alternative specification. Although the specific indirect association through MMSE alone was not statistically significant, several other indirect associations remained statistically non-zero. Accordingly, Model 81 neither validates the mediator ordering specified in Model 80 nor rules out alternative pathway structures. Instead, the findings indicate that more than one cross-sectional pathway structure is compatible with the observed data. Model 80 was retained as the primary model because it reflects the hypothesized conceptual framework of the study; nevertheless, the temporal ordering of the mediators cannot be determined from the present cross-sectional data.

### 4.4. Strengths, Limitations and Directions for Future Research

To our knowledge, the present study is among the first to examine a serial multiple-mediation model jointly including physical performance, depressive symptoms, cognitive status, functional status, and global self-rated health within the same geriatric inpatient sample. The analysis examined the statistical indirect associations between physical performance and EQ-VAS through depressive symptoms and cognitive status as parallel first-stage mediators, followed by functional status. By jointly modeling these variables, the study provides an integrated description of their cross-sectional associations and identifies psychological, cognitive, and functional domains that may be relevant to comprehensive geriatric assessment. The findings may also inform hypotheses for future longitudinal and intervention studies, but they do not establish causal mechanisms or demonstrate that modifying any of these variables would necessarily improve self-rated health.

The observed serial indirect associations were consistent with pathways in which depressive symptoms or cognitive status were associated with functional status, which, in turn, was associated with EQ-VAS-rated health. These pathways should not be interpreted as temporal or causal processes. These cross-sectional findings suggest that depressive symptoms, cognitive function, and functional independence merit consideration alongside physical performance in comprehensive geriatric assessment. Prospective studies are needed to examine the temporal relationships among these domains, while intervention studies should evaluate whether integrated assessment and care improve outcomes for vulnerable older adults in Romania. Such evidence could inform future healthy aging initiatives. The present study identified indirect associations between physical performance and EQ-VAS-assessed self-rated health in older patients. However, several limitations should be considered.

First, all study variables were assessed at a single time point. Consequently, the PROCESS estimates represent model-dependent cross-sectional indirect associations and cannot establish temporal precedence, directionality, or causal mediation. The ordering of depressive symptoms and cognitive status before functional status reflects the conceptual specification of Model 80 rather than an empirically demonstrated sequence. Methodological research has also shown that cross-sectional indirect-effect estimates may provide biased representations of longitudinal mediation processes [51]. The findings should therefore be regarded as exploratory and hypothesis-generating. Furthermore, all constructs were assessed during the same session and within the same clinical context, which may have introduced shared contextual influences and potential common method bias. However, the study combined different assessment modalities: TMT was a performance-based clinical assessment; MMSE and IADL were standardized cognitive and functional assessments; and GDS-15 and EQ-VAS relied on participants’ responses. This heterogeneity may reduce, but does not eliminate, the possibility that the associations were influenced by participants’ transient clinical or emotional state, fatigue, the hospitalization context, or assessor-related factors. Longitudinal studies with measurements collected at different time points are required to test the proposed ordering and evaluate potential causal mechanisms.

Second, the primary analysis dichotomized the TMT score at the boundary between the conventionally classified low-fall-risk range and the moderate/high-fall-risk ranges. Although this provided a clinically interpretable contrast, it reduced score information and combined two groups that may differ in the severity of balance and gait impairment. The binary results should therefore be interpreted as average differences between participants within versus below the low-fall-risk range rather than as evidence that the moderate- and high-risk groups are homogeneous. The sensitivity analysis using a continuous, reflected log-transformed TMT-derived predictor was included to assess the dependence of the findings on this coding decision.

Third, because this study was a secondary analysis of an existing cohort, no prospective sample-size calculation was conducted specifically for the serial multiple-mediation model. Consequently, the precision of estimates for small indirect associations may be limited, and non-significant indirect effects should be interpreted cautiously rather than as evidence of the absence of an association. Interpretation was therefore based on the estimated coefficients and their 95% bootstrap confidence intervals. Additionally, the dataset available for this secondary analysis contained total scores but not item-level responses for GDS-15, MMSE, and IADL. Consequently, sample-specific internal-consistency coefficients could not be estimated.

Fourth, although several confounding variables were adjusted for in the mediation models, other potential confounders, such as education, marital status, nutrition, chronic conditions, medication, and lifestyle characteristics (e.g., smoking, alcohol consumption, sleep), were unavailable or were not included in the analysis. Therefore, residual confounding cannot be excluded. For example, educational attainment, which may influence MMSE performance, was not available in the parent dataset. Moreover, a validated Charlson Comorbidity Index could not be reconstructed, and delirium, sarcopenia, and medication-specific anticholinergic burden were not assessed sufficiently or consistently. Sarcopenia may be associated with both mobility limitations and reduced IADL independence, while medication burden may affect mobility, cognitive performance, and daily functioning. These data constraints limited the scope for additional sensitivity analyses incorporating these potential confounders. Finally, the single-center Romanian inpatient sample limits generalizability to other clinical settings and populations.

Additionally, previous studies have suggested potential bidirectional relationships between the variables explored in this study. Therefore, alternative models are theoretically conceivable using different measurement instruments, including alternative orderings of the mediators, such as a serial multiple mediation model with three mediators (physical performance → depressive symptoms → cognitive status → functional status → health-related quality of life) or a serial-parallel mediation model (physical performance → functional status → depressive symptoms → cognitive status → health-related quality of life). The exploratory Model 81, in which functional status preceded depressive symptoms and cognitive status, also yielded several indirect associations with bootstrap confidence intervals excluding zero. This finding reinforces that the present cross-sectional data cannot distinguish among competing temporal structures. Longitudinal studies are needed to evaluate the temporal sequence of these associations and to compare theoretically plausible pathway models.

## 5. Conclusions

Using a cross-sectional serial multiple mediation model, this study identified statistically significant indirect associations between physical performance measured using the Tinetti Mobility test and EQ-VAS-assessed self-rated health through depressive symptoms, cognitive status, and functional status. Both single-mediator and serial association patterns were observed, although the serial association involving depressive symptoms and IADL was small and should be interpreted cautiously. The study’s results enhance our understanding of the associations between physical performance and EQ-VAS-assessed self-rated health in older adults and may inform future evaluations of integrated assessment and care strategies for older adults with low physical performance. These model-dependent findings support the joint assessment of physical, psychological, cognitive, and functional domains in older inpatients but do not establish temporal or causal pathways. Longitudinal studies are needed to test the proposed mediator ordering and determine whether these associations persist over time.

## Figures and Tables

**Table 1:** Demographic and clinical characteristics of patients (n = 287).

Characteristic	Category	Frequency (%)
Sex	Female	229 (79.8)
	Male	58 (20.2)
Age groups	65–74 years	145 (50.5)
	75–79 years	75 (26.1)
	≥80 years	67 (23.3)
Residence area	Urban	202 (70.4)
	Rural	85 (29.6)
Body mass index (BMI)	Not obese	190 (66.2)
	Obesity	97 (33.8)
Mini Nutritional Assessment (MNA)	Normal	226 (78.7)
	At risk of malnutrition	61 (21.3)
High blood pressure at assessment	No	269 (93.7)
	Yes	18 (6.3)
Smoking	No	224 (78.0)
	Yes	63 (22.0)
Alcohol use	No	197 (68.6)
	Yes	90 (31.4)
Chronic diseases		
Dyslipidemia	Yes	210 (73.2)
Atherosclerosis	Yes	72 (25.1)
Osteoarthritis	Yes	162 (56.4)

Note: For chronic diseases, only participants with the condition are shown; percentages were calculated using N = 287.

**Table 2:** Descriptive statistics and Spearman correlations of the main variables (n = 287).

Variable	n (%)	M (SD)	Correlations			
			TMT	GDS-15	MMSE	IADL
Tinetti mobility test (TMT)		23.30 (5.042)	1			
TMT groups						
Normal balance and gait (24–28)	150 (52.3)					
Fall risk present (19–23)	91 (31.7)					
High fall risk (0–18)	46 (16.0)					
Depressive symptoms (GDS-15)		3.25 (3.177)	−0.321 **	1		
GDS-15 groups						
Normal (0–4)	210 (73.2)					
Impaired (≥5)	77 (26.8)					
Mini-Mental State Examination (MMSE)		27.69 (3.013)	0.296 **	−0.292 **	1	
MMSE groups						
Normal	225 (78.4)					
Impaired	62 (21.6)					
Instrumental Activities of Daily Living (IADL)		7.27 (1.412)	0.580 **	−0.336 **	0.415 **	1
IADL groups						
Independent	197 (68.6)					
Dependent (moderate-severe)	90 (31.4)					
EQ Visual analogue scale (EQ-VAS)		86.92 (7.253)	0.558 **	−0.382 **	0.330 **	0.435 **

Abbreviations: n = number of patients; % = percentage; M = mean; SD = standard deviation. Note: ** *p* < 0.01.

**Table 3:** Results of regression analysis (n = 287).

Variable	EQ-VAS Without Mediators		EQ-VAS with Mediators		GDS-15		MMSE		IADL	
	β	t	β	t	β	t	β	t	β	t
TMT	0.794 ^1^	7.174	0.493 ^1^	5.118	−0.411 ^1^	−3.513	0.285 ^1^	2.228	0.461 ^1^	5.125
GDS-15	-	-	−0.181	−2.887	-	-	-	-	−0.095	−1.745
MMSE	-	-	0.270	2.414	-	-	-	-	0.456	4.135
IADL	-	-	0.238	2.743	-	-	-	-	-	-
Sex	−0.098	−1.536	−0.033	−0.706	−0.064	−1.053	−0.135	−1.798	−0.109	−1.960
Age_d2	−0.123	−1.910	0.038	0.590	0.136	1.901	−0.229	−2.991	−0.198	−3.057
Age_d3	−0.028	−0.432	−0.041	−0.690	−0.014	−0.214	0.038	0.653	−0.017	−0.322
Residence	0.056	1.170	0.105	2.103	−0.087	−1.561	−0.164	−2.824	−0.017	−0.324
Obesity	−0.060	−1.203	−0.099	−2.298	−0.035	−0.622	0.112	2.135	−0.044	−1.048
*R* ^2^	0.208		0.415		0.084		0.166		0.486	
*F*	11.641		11.445		4.172		7.128		16.001	

Abbreviations: TMT = physical performance measured by Tinetti mobility test (TMT), coded 0 = 0–23 and 1 = 24–28; GDS-15 = Geriatric Depression Scale; MMSE = Mini-Mental State Examination; IADL = Instrumental Activities of Daily Living; EQ-VAS = EuroQol Visual Analogue Scale; β = standardized regression coefficients; t = t statistic; R^2^ = coefficient of determination; F = F-test. ^1^ Coefficients for the dichotomous independent variable (TMT) are partially standardized (expressed in terms of the standard deviation of the outcome variable). Note: Age was entered as a categorical variable with three groups, coded 1 = 65–74 years (reference category), 2 = 75–79 years, 3 = 80 years and older. Age_d2 and Age_d3 are dummy variables.

**Table 4:** Chain mediation pathway analysis (n = 287).

	B	SE (HC4)	LLCI	ULCI
Total effect	5.761	0.803	4.180	7.341
Total direct effect	3.576	0.699	2.200	4.952
	B	BootSE	Bootstrap 95% CI	
			BootLLCI	BootULCI
Total indirect effect	2.184	0.605	1.118	3.493
Ind1: TMT → GDS-15 → EQ-VAS	0.540	0.231	0.170	1.064
Ind2: TMT → MMSE → EQ-VAS	0.557	0.347	0.048	1.373
Ind3: TMT → IADL → EQ-VAS	0.796	0.332	0.218	1.510
Ind4: TMT → GDS-15 → IADL → EQ-VAS	0.067	0.050	0.001	0.191
Ind5: TMT → MMSE → IADL → EQ-VAS	0.224	0.142	0.016	0.554
(C1) Ind1–Ind2	−0.018	0.414	−0.897	0.718
(C2) Ind1–Ind3	−0.256	0.426	−1.079	0.587
(C3) Ind1–Ind4	0.473	0.223	0.117	0.983
(C4) Ind1–Ind5	0.315	0.277	−0.185	0.914
(C5) Ind2–Ind3	−0.239	0.516	−1.178	0.861
(C6) Ind2–Ind4	0.490	0.358	−0.037	1.328
(C7) Ind2–Ind5	0.333	0.283	−0.032	1.034
(C8) Ind3–Ind4	0.729	0.320	0.181	1.403
(C9) Ind3–Ind5	0.572	0.300	0.084	1.232
(C10) Ind4–Ind5	−0.157	0.147	−0.489	0.072

Abbreviations: TMT = physical performance measured by Tinetti mobility test (TMT); GDS-15 = Geriatric Depression Scale; MMSE = Mini-Mental State Examination; IADL = Instrumental Activities of Daily Living; EQ-VAS = EuroQol Visual Analogue Scale; B = unstandardized coefficient; BootSE = Bootstrap Standard Error; BootLLCI = lower limit of the bootstrap confidence interval; BootULCI = upper limit of the bootstrap confidence interval; CI = Confidence Interval; Note: Statistical inference for each specific indirect association was based on whether its 95% bootstrap confidence interval excluded zero; statistical significance of every individual component path was not required.

## Data Availability

The dataset analysed in this article is not publicly available because it is owned by the institution that conducted the study.

## Supplementary Materials

The following supporting information can be downloaded at: https://www.mdpi.com/article/10.3390/healthcare14203390/s1, Table S1: Sensitivity analysis using PROCESS Model 80 with TMT as a continuous predictor; Table S2: Sensitivity analysis using PROCESS Model 81 with an alternative ordering of the mediators.
