# Supplementary material for: Serial Mediation Pathways Linking Physical Performance to EQ-VAS-Assessed Self-Rated Health in Older Inpatients
Source: Healthcare (Basel). 2026 Oct 12;14(20):3390. doi: 10.3390/healthcare14203390 (PMC13704198; doi:10.3390/healthcare14203390)
Supplement: Supplementary file 1 [file healthcare-14-03390-s001.zip › healthcare-4571513-supplementary.pdf]

Supplementary Material

Table S1. **Sensitivity analysis using PROCESS Model 80 with TMT as a continuous predictor**

===

************** TOTAL, DIRECT, AND INDIRECT EFFECTS OF X ON Y **************

Total effect of X on Y

Effect	se(HC4)	t	p	LLCI	ULCI	c_cs
-8.6985	1.1163	-7.7922	.0000	-10.8960	-6.5011	-.4962

Direct effect of X on Y

Effect	se(HC4)	t	p	LLCI	ULCI	c'_cs
-5.3208	.9802	-5.4280	.0000	-7.2504	-3.3911	-.3035

Indirect effect(s) of X on Y:

	Effect	BootSE	BootLLCI	BootULCI
TOTAL	-3.3778	.8773	-5.2663	-1.8423
Ind1	-.8263	.3441	-1.6075	-.2650
Ind2	-1.0830	.5741	-2.4125	-.2450
Ind3	-1.0754	.5006	-2.1269	-.1669
Ind4	-.0583	.0611	-.2089	.0304
Ind5	-.3347	.2059	-.8257	-.0406
(C1)	.2567	.7131	-.9846	1.7688
(C2)	.2491	.6411	-1.0321	1.4880
(C3)	-.7680	.3448	-1.5495	-.1891
(C4)	-.4916	.4284	-1.4109	.3017
(C5)	-.0076	.8170	-1.7605	1.4539
(C6)	-1.0247	.5892	-2.3973	-.1496
(C7)	-.7483	.5124	-1.9922	-.0251
(C8)	-1.0171	.4886	-2.0498	-.1534
(C9)	-.7407	.4136	-1.6564	-.0696
(C10)	.2764	.2114	-.0243	.7789

===

Supplementary Material

Table S2. **Sensitivity analysis using PROCESS Model 81 with an alternative ordering of the mediators**

===

************** TOTAL, DIRECT, AND INDIRECT EFFECTS OF X ON Y **************

Total effect of X on Y

Effect	se(HC4)	t	p	LLCI	ULCI	c_ps
5.7605	.8029	7.1744	.0000	4.1800	7.3410	.7942

Direct effect of X on Y

Effect	se(HC4)	t	p	LLCI	ULCI	c'_ps
3.5762	.6988	5.1175	.0000	2.2005	4.9518	.4931

Indirect effect(s) of X on Y:

	Effect	BootSE	BootLLCI	BootULCI
TOTAL	2.1843	.6083	1.1401	3.4960
Ind1	1.0875	.4303	.3014	2.0054
Ind2	.3677	.1952	.0528	.8099
Ind3	-.1278	.2256	-.6008	.2974
Ind4	.1719	.0882	.0365	.3776
Ind5	.6850	.3549	.1644	1.4972
(C1)	.7197	.4806	-.1797	1.7023
(C2)	1.2153	.5016	.2736	2.2093
(C3)	.9156	.4490	.0806	1.8429
(C4)	.4025	.5235	-.6726	1.3854
(C5)	.4956	.2725	-.0041	1.0628
(C6)	.1958	.2025	-.1611	.6518
(C7)	-.3173	.3972	-1.1693	.3759
(C8)	-.2997	.2325	-.7769	.1502
(C9)	-.8128	.4808	-1.9092	-.0897
(C10)	-.5131	.3908	-1.3915	.0910

===
